# Acetylcholinesterase from Human Erythrocytes as a Surrogate Biomarker of Lead Induced Neurotoxicity

**DOI:** 10.1155/2015/370705

**Published:** 2015-10-22

**Authors:** Vivek Kumar Gupta, Rajnish Pal, Nikhat Jamal Siddiqi, Bechan Sharma

**Affiliations:** ^1^Department of Biochemistry, Faculty of Science, University of Allahabad, Allahabad 211002, India; ^2^Department of Biochemistry, College of Science, King Saud University, P.O. Box 22452, Riyadh 11495, Saudi Arabia

## Abstract

Lead induced neurotoxicity in the people engaged in different occupations has received wide attention but very little studies have been carried out to monitor occupational neurotoxicity directly due to lead exposure using biochemical methods. In the present paper an endeavour has been made in order to assess the lead mediated neurotoxicity by *in vitro* assay of the activity of acetylcholinesterase (AChE) from human erythrocytes in presence of different concentrations of lead. The results suggested that the activity of this enzyme was localized in membrane bound fraction and it was found to be highly stable up to 30 days when stored at −20°C in phosphate buffer (50 mM, pH 7.4) containing 0.2% Triton X-100. The erythrocyte's AChE exhibited *K*_*m*_ for acetylcholinesterase to be 0.1 mM. Lead caused sharp inhibition of the enzyme and its IC_50_ value was computed to be 1.34 mM. The inhibition of the enzyme by lead was found to be of uncompetitive type (*K*_*i*_ value, 3.6 mM) which negatively influenced both the *V*_max_ and the enzyme-substrate binding affinity. Taken together, these results indicate that AChE from human erythrocytes could be exploited as a surrogate biomarker of lead induced neurotoxicity particularly in the people occupationally exposed to lead.

## 1. Introduction

There are concrete evidences to prove that numerous environmental toxicants, including lead, have potential to induce neurotoxicity by their strong interference in the neurotransmission function of the central nervous system [1, 2]. Lead nitrate is an inorganic compound with the chemical formula Pb(NO_3_)_2_. Lead (Pb; Latin: plumbum, atomic number 82) is another ubiquitous toxic metal detectable practically in all phases of the inert environment and biological systems [3–5]. Lead is frequently used in the production of batteries, metal products (solder and pipes), ammunition, and devices to shield X-rays leading to its exposure to the people working in these industries. Use of lead in gasoline, paints and ceramic products, caulking, and pipe solder has been dramatically reduced in recent years because of health concerns. The most susceptible population to lead poisoning is children, particularly soldiers, infants in neonatal periods, and the fetus [6–9]. Lead poisoning (also known as saturnism, plumbism, Devon colic, or painter's colic) is a medical condition caused by increased levels of lead in the blood [10].

This metal does not have any known useful function in the human body and produces harmful effects once it enters the body through inhalation, ingestion, and skin contact [11]. Lead replaces other useful divalent metal ions involved in key physiological functions of the body and interferes with the cholinergic systems resulting in impairments in central nervous system including cognitive behavior [12, 13]. Lead in the chemical form of lead nitrate is a potential oxidant and recognized as a carcinogenic element to humans. Due to its hazardous nature, the limited applications of lead nitrate are under constant scrutiny. Other industrial applications of lead included heat stabilization in nylon and polyesters, and in coatings of photothermographic paper. Around the year 2000, lead nitrate has begun to be used in gold cyanidation. Lead is generally more toxic and it binds readily to thiol groups of proteins/peptides and tends to disrupt their functions by inactivation or precipitation [14].

Acetylcholinesterase (AChE; EC 3.1.1.7), also known as AChE or acetylhydrolase, is a hydrolase that catalyses hydrolysis of the neurotransmitter, acetylcholine (Ach). AChE is found mainly at neuromuscular junctions and cholinergic brain synapses, where its activity serves to terminate synaptic transmission. It belongs to carboxyl esterase family of enzymes. It is synthesized in the endoplasmic reticulum and is then exported towards the cellular surface, where its different molecular/globular forms may be anchored in plasma membrane, attached to the basal lamina (asymmetric collagen-tailed forms), or secreted as soluble molecules (nonglobular) forms [15]. It is reported that dimeric (G) AChE forms are present in the human erythrocytes [16]. Erythrocyte AChE has been found to be firmly attached to the membrane components and therefore it is more difficult to solubilize than brain [17]. The most widely adopted solubilization methods for mammalian brain AChE have involved the application of detergents, particularly Triton X-100, a nonionic detergent [18]. AChE has been widely exploited as a primary target of action by organophosphorus compounds such as nerve agents [19]. As a reliable indicator, it is used in the diagnosis of poisoning caused by reversible and irreversible inhibitors including heavy metals and pesticides. The proper binding mechanism of the ACh with AChE has been well documented by kinetics as well as molecular modeling studies with different inhibitors.

Lead induced neurotoxicity in the people engaged in different occupations and exposed to this heavy metal has received wide attention but very little studies have been carried out to monitor occupational neurotoxicity directly using biochemical methods. Since AChE in erythrocytes is one of the typical extra neural AChE enzymes and exhibits similarities in its several properties with that of neuronal enzyme, we have used it as a surrogate biomarker to assess the lead mediated neurotoxicity and the mechanism of its action on the enzyme* in vitro*. In present study, the enzyme has been characterized to demonstrate its cellular localization and biophysical and biochemical properties with special reference to interaction of lead with enzyme protein leading to alterations in its biochemical functions.

## 2. Materials and Methods

### 2.1. Chemicals

S-Acetylthiocholine iodide (ATI) was from Tokyo Chemical Industry Co., Ltd., Tokyo, Japan and the colouring reagent 5,5′-dithio-bis(2-nitrobenzoic acid) (DTNB) was from SRL Pvt. Ltd., Mumbai, India. Triton X-100 was procured from Merck. Bovine serum albumin, phosphate buffer salts (sodium dihydrogen orthophosphate and disodium hydrogen phosphate) were obtained from Fisher Scientific and Folin and Ciocalteu's Phenol reagent was from Spectrochem Pvt. Ltd., Mumbai, India. All other chemicals were commercial products of analytical grade purity.

### 2.2. Preparation of Ghosts

Human blood sample was collected from a healthy 24-year-old male not exposed to radiation, drugs, or any other antioxidant supplementation, including vitamins, by venipuncture, into an EDTA coated vial and was centrifuged at 3000 rpm for 5 min at 4°C for the removal of plasma. The erythrocytes were washed thrice with cold 2x phosphate buffer saline (20 mM phosphate buffer containing 274 mM NaCl and 5.4 mM KCl; pH 7.4) and centrifuged. After that, the packed erythrocytes were resuspended in hypotonic phosphate buffer of 5 mM Tris HCl containing 1 mM EDTA (pH 7.4) and kept at 10°C for overnight incubation. Then the erythrocytes were centrifuged for 7-8 times with the hypotonic phosphate buffer at 9000 rpm for 10 min at 4°C to get the ghost (erythrocyte membranes) at the bottom of the tube. This preparation (ghost cells after the loss of haemoglobin) was treated with Triton X-100 (0.2%, v/v; prepared in phosphate buffer 50 mM, pH 7.4) to solubilize membrane bound enzyme, AChE.

### 2.3. AChE Extraction

The ghost cells, after the loss of haemoglobin, were treated with Triton X-100 (0.2%, v/v; prepared in phosphate buffer 50 mM pH 7.4) to solubilize membrane bound enzyme, that is, AChE. Human Ghost (as described above) was homogenized by applying 3 strokes, each of 30 sec in the presence of precooled 50 mM phosphate buffer (pH 7.4) containing 0.2% Triton X-100 to produce homogenate. The homogenate was centrifuged at 10,000 g for 10 min at 4–6°C and the clear supernatant was used for the AChE assays.

### 2.4. Protein Estimation

Protein was estimated by the Folin and Ciocalteu's Phenol reagent [20] using bovine serum albumin as a standard. The absorbance of blue coloured complex was measured at 620 nm.

### 2.5. Acetylcholinesterase Assay

The AChE activity was assayed by following the method of Ellman et al. (1961) [21]. The reaction mixture (3 mL) in quartz cuvette having 1 cm path length contains 0.50 mM of ATI, 0.5 mM of DTNB, and 50 mM phosphate buffer (pH 7.4). The change in optical density was measured at 412 nm for 3 min at each interval of 30 sec. The AChE activity was calculated using extinction coefficient 13.6 × 10^3^ M^−1 ^cm^−1^ and expressed as *μ*moles of acetylthiocholine (ATI) hydrolyzed mL^−1 ^min^−1^ or units (U). The specific activity of enzyme was expressed in U mg^−1^. The enzyme assays were performed on UV-Visible double beam spectrophotometer (Thermo Scientific Spectroscan UV 2700). The catalytic activity is measured by the increase of the yellow anion, 5-thio-2-nitrobenzoate, produced due to reaction of thiocholine with 5,5′-dithio-bis-(2-nitrobenzoic acid) (DTNB). The assay system without substrate or enzyme was considered as a substrate or enzyme blank, respectively, and any change in absorbance min^−1^ recorded in this condition was subtracted from the experimental observations.

### 2.6. Effect of Substrate Concentration on AChE Activity

The kinetic parameters such as Michaelis-Menten constant (*K*_*m*_) and maximum velocity (*V*_max_) were estimated by assaying the enzyme activity using varying substrate (acetylthiocholine iodide, ATI) concentrations (0 to 2 mM) and fixed enzyme protein (50 *μ*g) at room temperature (26 ± 2°C).

### 2.7. Determination of Effect of Time on Lead Mediated Inhibition of AChE

The enzyme (50 *μ*g) was assayed in the presence of 0.25 mM lead at varying time periods (0 to 120 min) at room temperature (26 ± 2°C). The residual enzyme activity was monitored. The activity of enzyme was also recorded at these time points in absence of lead, which served as a control. The reaction rate measured soon after mixing the enzyme with other reagents without any further incubation was used as zero time reaction. The data of percent residual activity and the time of incubation in min were extrapolated at *y*-axis and *x*-axes, respectively. The *t*_1/2_ value (the time at which the enzyme activity remains half of the original under this condition) was calculated from this plot.

### 2.8. Estimation of IC_50_ Value for Lead

The enzyme (50 *μ*g) was assayed in the presence of different concentrations of lead nitrate and the residual activity was monitored. The activity recorded in absence of lead was considered as 100%. The IC_50_ value was calculated by extrapolating the data taking percent residual activity on *y*-axis and the varying lead concentrations at *x*-axis on a graph.

### 2.9. Determination of Mode of Inhibition of AChE by Lead

The enzyme (50 *μ*g) was assayed at varying concentrations of ATI at room temperature (26 ± 2°C) in the absence and presence of lead (0.5 mM). The *K*_*i*_ and *V*_max_ values were calculated using the intersections by the straight line at *y*-axis and at the negative abscissa of *x*-axes, respectively, of the Lineweaver-Burk's double reciprocal plot.

### 2.10. Determination of *K*_*i*_ Value for Lead in Uncompetitive Mode of Inhibition of AChE from Human Erythrocytes

The mode of inhibition of enzyme by lead was determined by assaying the enzyme mentioned as above using the formula of either *V*_max+1_ = *V*_max−1_/1 + [I]/*K*_*i*_ or *K*_*m*+1_ = *K*_*m*_/1 + [I]/*K*_*i*_ where *V*_max+1_ and *V*_max−1_ are the maximal velocities of reactions in the presence and absence of lead. Similarly, *K*_*m*+1_ and *K*_*m*_ denote the *K*_*m*_ values in the presence and absence of lead. [I] represents the concentration of inhibitor used, that is, 0.5 mM. The *K*_*i*_ value may also be calculated using *K*_*i*_ = IC_50_/1 + [S]/*K*_*m*_.

### 2.11. Statistical Analysis of Data

Statistical analysis of data was performed using Graph Pad Prism version 6 for windows. All values were expressed as mean standard deviation of 3 different observations.

## 3. Results

### 3.1. Membrane Bound Nature of AChE from Human Erythrocytes

The enzyme from the ghosts was solubilized using a nonionic detergent, 0.2% (v/v) Triton X-100 in phosphate buffer (50 mM, pH 7.4). The extent of enzyme activity was 10 times more in the detergent solubilized fraction than that without detergent. These results demonstrated the membrane bound nature of this enzyme. The protein contents in the soluble fractions of these two preparations were also found to be significantly different. The fraction obtained with Triton X-100 contained 2.60 mg/mL protein as against 0.9 mg/mL in the fraction without treatment with the detergent.

### 3.2. Stability of Enzyme Activity after Storage at −20°C

The effect of storage time at −20°C on the activity of AChE from human erythrocytes was determined by carrying out the enzyme assay employing 50 *μ*g protein on different days as described in Materials and Methods. The enzyme was stored in phosphate buffer (50 mM, pH 7.4) containing 0.2% (v/v) Triton X-100. The results shown in Table 1 indicated that the enzyme was highly stable up to 30 days with much loss in activity. However, when this enzyme was assayed at varying temperatures, it exhibited maximum activity at 37°C followed by gradual loss in its activity after increasing temperature further (data not shown). The enzyme was found to be optimally active at pH 7.4 when assayed using buffers of different pH systems under standard assay conditions (data not shown). The buffers of higher pH values displayed exerting inhibitory effect on enzyme activity.

### 3.3. Effect of Substrate Concentration on the Activity of AChE from Human Erythrocytes

**Figure 1:**
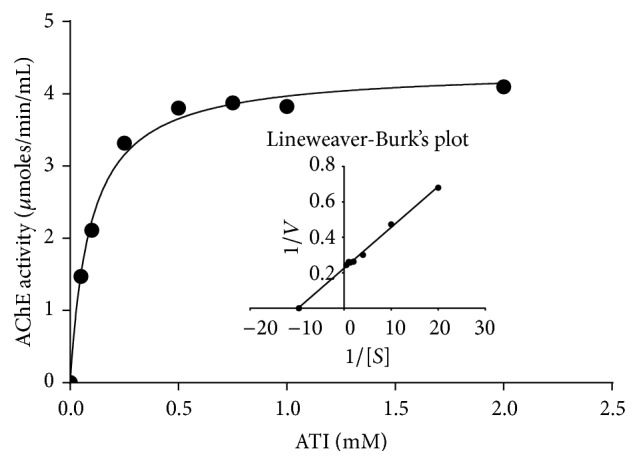
Effect of substrate (ATI) on the activity of AChE from human erythrocytes was observed by assaying the enzyme at varying concentration ATI at room temperature (26 ± 2°C) as described in Materials and Methods employing 50 *μ*g protein. The *K*_*m*_ and *V*_max_ values were calculated using the intersection of the straight line at *y*-axis and at the negative abscissa on *x*-axis, respectively.

The enzyme (50 *μ*g) was assayed at varying concentrations of the substrate, ATI, at room temperature (26 ± 2°C). The enzyme activity at corresponding substrate concentration displayed a direct correlation and the results showed a hyperbolic curve (data not shown). The Lineweaver-Burk's double reciprocal plot of the data as shown in Figure 1 demonstrated a straight line which intersects at *y*-axis and negative abscissa of *x*-axes, from where the *V*_max_ and *K*_*m*_ values could be calculated and the values being 4.04 *μ*moles mL^−1 ^min^−1^ and 0.1 mM, respectively.

### 3.4. Effect of Lead on the Activity of AChE from Human Erythrocytes

**Figure 2:**
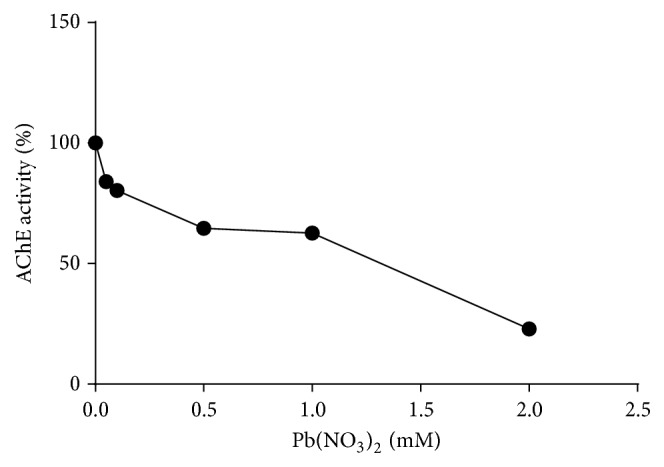
Determination of IC_50_ value of lead for human erythrocyte AChE using the data from Table 2.

The enzyme when assayed in presence of varying concentrations of lead (0–2 mM) displayed consistent decrease in its activity (Table 1). When this data was extrapolated using percent residual activity and the lead concentrations on *y*-axis and *x*-axes, respectively, the IC_50_ value of this heavy metal for erythrocytes AChE could be determined, the value being 1.34 mM (Figure 2 [Table tab2]).

### 3.5. Effect of Time on Lead Mediated Inhibition of AChE Activity from Human Erythrocytes

**Figure 3:**
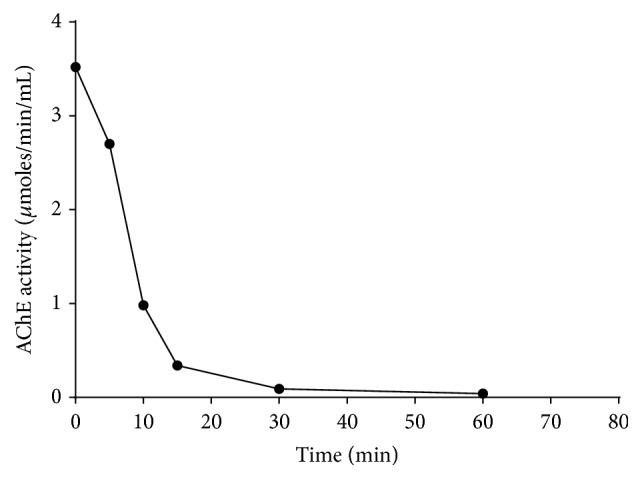
Effect of lead (0.25 mM) on the activity of AChE from human erythrocytes with respect to the varying incubation time at room temperature (26 ± 2°C). The enzyme assay was carried out employing 50 *μ*g protein using the procedure as described in Materials and Methods. The results indicate the average values of three independent experiments. The enzyme in the absence of lead served as a control and did not show any decrease in activity.

The effect of time on the lead mediated inhibition of the AChE activity from human erythrocytes at room temperature (26 ± 2°C) was monitored by assaying the enzyme (50 *μ*g) at different time intervals in absence and presence of the heavy metal (0.25 mM). The enzyme activity in the absence of lead served as a control. The results as presented in Figure 3 demonstrated that the enzyme activity decreased consistently with respect to the increasing incubation time in presence of lead. The results from present study also demonstrated that lead at a concentration of 0.25 mM caused sharp inhibition of the enzyme in a time dependent manner at 26 ± 2°C temperature. The enzyme activity remained only about 40% after 10 min of incubation. The time taken to lose about 50% activity of this enzyme (*t*_1/2_) under this condition was about 8 min. However, the enzyme did not show any decrease in activity in the absence of lead under similar experimental conditions.

### 3.6. Evaluation of Mode of Inhibition of AChE from Human Erythrocytes by Lead

**Figure 4:**
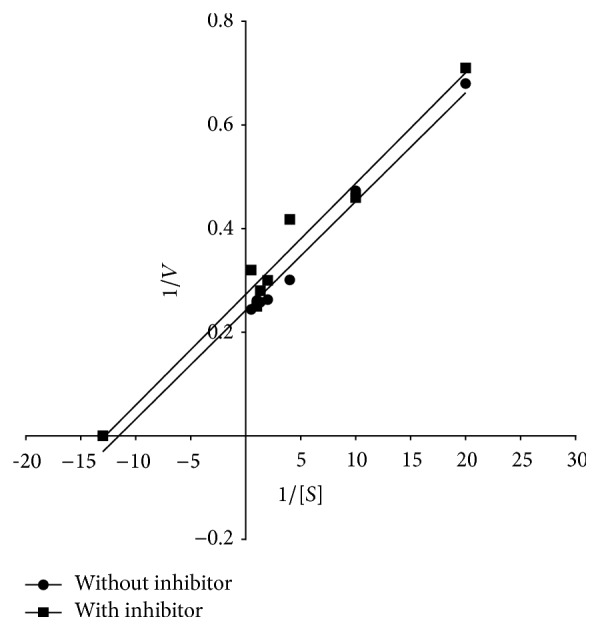
Determination of mechanism of AChE inhibitory action of lead. The enzyme (50 *μ*g) was assayed at varying concentration ATI at room temperature (26 ± 2°C) in the absence (●) and presence (■) of lead (0.5 mM) as described in Materials and Methods. The *K*_*i*_ and *V*_max_ values were calculated using the intersections of the straight line at *y*-axis and at the negative abscissa on *x*-axes, respectively.

The above experiments indicated that treatment of erythrocytes AChE with lead resulted in sharp decline in its activity. In order to ascertain the mechanism of inhibition of AChE activity by lead, the enzyme (50 *μ*g) was assayed at varying substrate concentrations in absence and presence of lead (0.5 mM). The data were used to extrapolate Lineweaver-Burk's double reciprocal plot, which developed two straight lines parallel to each other intersecting at different points on *y*-axis and *x*-axes, respectively. The *V*_max+1_ (*V*_max_ value in presence of lead) and *K*_*i*_ values were calculated using these points and found to be 3.703 *μ*moles/mL/min and 3.6 mM, respectively. The results are shown in Figure 4.

## 4. Discussion

AChE is responsible for transmission interruption of normal nerve transmission at the synapse by hydrolyzing the neurotransmitter acetylcholine (Ach) to acetic acid and choline in order to avoid undesired stimulation of nervous system. The reports on the presence of lead and its adverse effects in the occupants exposed to this heavy metal while working in various lead infested environments make it imperative to study the assessment of the impact of lead on the activity of AChE responsible for regulating the cholinergic functions and physiological activities of humans [2, 22, 23]. Continuously for about two decades in past from now the studies on dose-response relationship concerning lead mediated neurotoxicity were carried out using only neurobehavioral tests [24] or electrophysiological measurements [25] on the pretext of the unavailability of the cells or tissues of human's central nervous system, lack of sensitive neurochemical indicators, and also the ethical considerations. The peripheral tissues such as erythrocytes and other blood cells exhibiting some components of neurotransmission such as AChE and Na^+^, K^+^ ATPases from erythrocytes, adrenergic and muscarinic receptors from lymphocytes, and adrenergic receptors from platelets [26, 27], however, were found to act as the most viable alternatives to study lead mediated neurotoxicity in humans using direct biochemical methods without facing a lot of hassles as mentioned above.

In this study, the activity of AChE was determined by extraction of the enzyme protein from human erythrocytes. This enzyme has been extracted by other workers using other detergents as well as low concentration of NaCl (0.14 M) [28]. They have shown that both hydrophobic and electrostatic forces are involved in binding of the AChE to human erythrocyte membrane. NaCl probably weakens the electrostatic bonding with membrane proteins that shields some of the enzyme from the action of the detergent, and in this way NaCl allows a more effective attack of the detergent on the membrane to solubilize more membrane bound acetylcholinesterase. Moreover, some AChE was not released from the membrane even by using the Triton X-100 as well as mixtures of Triton X-100 and NaCl, suggesting that either it is more tightly bound to the membrane or it is shielded with such layers of macromolecules which are ineffective towards the action of Triton X-100 and NaCl although its amount was much less. AChE from erythrocytes exhibited maximum activity at pH 7.4 and 37°C temperature. Similar observations have also been reported by other workers [28, 29]. This enzyme exhibited Michaelis-Menten constant (*K*_*m*_) value for ATI to be 0.1 mM which was quite close to that reported by other workers for the AChE of blood cells isolated from human erythrocytes [30] as well as from other animals [31].

The IC_50_ value of lead for erythrocytes AChE in present study was recorded to be 1.34 mM. The effect of many other heavy metals such as mercury, cadmium, copper, and lead on the activity of human erythrocytes AChE has been reported recently by Ademuyiwa et al. [2] in the people who were consistently exposed to lead during different occupations. The estimation of IC_50_ value of lead for AChE of human erythrocytes, however, has not been conducted* in vitro* by any other worker elsewhere. The results from present study also demonstrated that lead at a concentration of 0.25 mM caused sharp decrease in the enzyme activity in time dependent manner at room temperature (26 ± 2°C), the *t*_1/2_ value being 8 min. No such studies have been carried out by other workers elsewhere to assess the time dependent inhibition of AChE by lead* in vitro*.

Lead displayed inhibition of erythrocytes AChE in uncompetitive manner with *K*_*i*_ value being 3.6 mM, thereby reducing both the *V*_max_ and substrate binding affinity of the enzyme. As shown in the following reaction scheme, lead as an uncompetitive inhibitor [I] has opportunity only to reversibly combine with the enzyme [E]-substrate [S] complex [ES] and not to the free enzyme. Lead is negatively influencing both the *V*_max_ and the *K*_*m*_ values for the enzyme. The decrease in *V*_max_ values occurs because some of the substrate is always bound in ESI complexes where it cannot be converted into product, decreasing the effective dissociation constant for the substrate. It results in a concomitant decrease in the *K*_*m*_ value as well. Since the *V*_max_ and the *K*_*m*_ are decreased by the same factor, the ratio of *K*_*m*_/*V*_max_ remains the same and gives rise to two parallel lines corresponding to the uninhibited and inhibited reactions in Lineweaver-Burk's double reciprocal plot. No such studies have been carried out by other workers elsewhere:

(1) $$\begin{array}{c} E+S\overset{+S}{⟺}\underset{\begin{array}{c} -I↑↓+I\\ ESI \end{array}}{ES}⟹E+P \end{array}$$

The reaction scheme shows mechanism of uncompetitive inhibition of enzyme. The −I and +I indicate the dissociation of inhibitor from ESI complex and association of inhibitor with ES complex, respectively.

Lead is known to exert its action in the biological systems through binding with the sulfhydryl groups of the proteins [32, 33]. AChE from human erythrocytes does not contain any free thiol group for this purpose. Thus, the strong inhibition of this enzyme by lead as observed in the present study is noteworthy. The uncompetitive mode of inhibition by lead indicates that it has some interaction sites either in the catalytic pocket or on the surface of the enzyme protein, which needs to be ascertained by further detailed investigation using suitable bioinformatics tools.

## 5. Conclusion

The AChE present in membrane bound forms in the human erythrocyte could be solubilized employing a nonionic detergent, Triton X-100. The drastic inhibition of AChE activity in human erythrocyte by lead nitrate suggests that the compound even at low concentration (*K*_*i*_ 3.6 mM) is extremely neurotoxic to the mammals. The striking feature of lead was its potential to decrease enzyme activity in time dependent manner as the presence of its 0.25 mM concentration resulted in loss of 60% enzyme activity in just 10 min. Though the exact mechanism of action of lead on erythrocytes AChE is not known by this study it is evident that this heavy metal inhibits the enzyme in an uncompetitive manner and hence adversely influences both the optimum velocity of reaction (*V*_max_) and the extent of enzyme-substrate binding affinity (*K*_*m*_) together. Thus, the results of this study as well as those available from other workers suggest that AChE from human erythrocytes might be exploited as a putative indicator to monitor lead induced perturbations in the cholinergic system of human population actively occupied in lead infested environment.

## Figures and Tables

**Table 1:** Stability of rat brain AChE activity.

S. number	Days	% AChE activity remaining
1	0	100
2	7	100
3	14	99.8
4	21	99.8
5	28	99.5
6	30	99.5

Effect of storage time at −20°C on the activity of AChE from human erythrocytes was observed by carrying out the enzyme assay employing 50 *µ*g protein on different days as described in Materials and Methods. The enzyme was stored in phosphate buffer (50 mM, pH 7.4) containing 0.2% Triton X-100.

**Table 2 tab2:** Effect of lead on the activity of AChE from human erythrocytes.

S. number	Lead concentration (mM)	Activity (*μ*mol/mL/min)	% AChE activity remaining
1	0.00	2.992	100.00
2	0.05	2.514	84.02
3	0.10	2.404	80.34
4	0.50	1.933	64.60
5	1.00	1.875	62.67
6	2.00	0.683	22.83

The effect of varying concentrations of lead on the activity of AChE from human erythrocytes has been determined. The enzyme assay was carried out employing 50 *µ*g protein using the procedure as described in Materials and Methods. The enzyme without lead served as a control and was considered to have 100% activity.

## References

[1] Nehru B., Sidhu P. (2001). Behavior and neurotoxic consequences of lead on rat brain followed by recovery. *Biological Trace Element Research*.

[2] Ademuyiwa O., Ugbaja R. N., Rotimi S. O. (2007). Erythrocyte acetylcholinesterase activity as a surrogate indicator of lead-induced neurotoxicity in occupational lead exposure in Abeokuta, Nigeria. *Environmental Toxicology and Pharmacology*.

[3] Qureshi N., Sharma R. (2012). Lead toxicity and infertility in female Swiss mice: a review. *Journal of Chemical, Biological and Physical Sciences*.

[4] Ghasemi H., Rostampour F., Ranjbar A. (2013). The role of oxidative stress in metals toxicity/mitochondrial dysfunction as a key player. *Galen Medical Journal*.

[5] Nava-Ruíz C., Méndez-Armenta M. (2013). Cadmium, lead, thallium: occurrence, neurotoxicity and histopathological changes of the nervous system. *Pollutant Diseases, Remediation and Recycling*.

[6] Boucher O., Burden M. J., Muckle G. (2012). Response inhibition and error monitoring during a visual Go/No-Go task in Inuit children exposed to lead, polychlorinated biphenyls, and methylmercury. *Environmental Health Perspectives*.

[7] Vallascas E., de Micco A., Deiana F., Banni S., Sanna E. (2013). Adipose tissue: another target organ for lead accumulation? A study on Sardinian children (Italy). *American Journal of Human Biology*.

[8] Li Y., Wu S., Xiang Y., Liang X. (2014). An investigation of outpatient children's blood lead level in Wuhan China. *PLoS ONE*.

[9] Pulido M. D., Parrish A. R. (2003). Metal-induced apoptosis mechanisms. *Mutation Research*.

[10] Liu C., Huo X., Lin P., Zhang Y., Li W., Xu X. (2015). Association between blood erythrocyte lead concentrations and hemoglobin levels in preschool children. *Environmental Science and Pollution Research*.

[11] Diamond G. L. (2005). Risk assessment of nephrotoxic metals. *The Toxicology of the Kidney*.

[12] Manzo L., Castoldi A. F., Coccini T., Rossi A. D., Nicotera P., Costa L. G. (1995). Mechanisms of neurotoxicity: applications to human biomonitoring. *Toxicology Letters*.

[13] Sharma B., Singh S., Siddiqi N. J. (2014). Biomedical implications of heavy metals induced imbalances in redox systems. *BioMed Research International*.

[14] Rogers L. E., Battles N. D., Reimold E. W., Sartain P. (1971). Erythrocyte enzymes in experimental lead poisoning. *Archiv für Toxikologie*.

[15] Chatel J.-M., Vallette F.-M., Massoulié J., Grassi J. (1993). A conformation-dependent monoclonal antibody against active chicken acetylcholinesterase. *FEBS Letters*.

[16] Ott P., Brodbeck U. (1978). Multiple molecular forms of acetylcholinesterase from human erythrocyte membranes. *European Journal of Biochemistry*.

[17] Massoulié J., Bon S. (1982). The molecular forms of cholinesterase and acetylcholinesterase in vertebrates. *Annual Review of Neuroscience*.

[18] Rieger F., Vigny M. (1976). Solubilisation and physicochemical characteriusation of rat brain acetylcholinesterase: development and maturation of its molecular forms. *Journal of Neurochemistry*.

[19] Pohanka M. (2011). Cholinesterases, a target of pharmacology and toxicology. *Biomedical Papers Olomouc*.

[20] Lowry O. H., Rosebrough N. J., Farr A. L., Randall R. J. (1951). Protein measurement with the Folin phenol reagent. *The Journal of Biological Chemistry*.

[21] Ellman G. L., Courtney K. D., Andres V., Featherstone R. M. (1961). A new and rapid colorimetric determination of acetylcholinesterase activity. *Biochemical Pharmacology*.

[22] Dosumu O., Onunkwor B., Odukoya O., Arowolo T., Ademuyiwa O. (2005). Biomarkers of lead exposure in auto-mechanics in Abeokuta, Nigeria. *Trace Elements and Electrolytes*.

[23] Lionetto M. G., Caricato R., Calisi A., Giordano M. E., Schettino T. (2013). Acetylcholinesterase as a biomarker in environmental and occupational medicine: new insights and future perspectives. *BioMed Research International*.

[24] Anger W. K. (1990). Worksite behavioral research. Results, sensitive methods, test batteries and the transition from laboratory data to human health. *Neurotoxicology*.

[25] Seppalainen A. M. H. (1988). Neurophysiological approaches to the detection of early neurotoxicity in humans. *CRC Critical Reviews in Toxicology*.

[26] Costa L. G., Manzo L. (1995). Biochemical markers of neurotoxicity: research strategies and epidemiological applications. *Toxicology Letters*.

[27] Manzo L., Artigas F., Martínez E. (1996). Biochemical markers of neurotoxicity. A review of mechanistic studies and applications. *Human and Experimental Toxicology*.

[28] Civenni G., Test S. T., Brodbeck U., Bütikofer P. (1998). In vitro incorporation of GPI-anchored proteins into human erythrocytes and their fate in the membrane. *Blood*.

[29] Marcos M. R., Sánchez-Yagüe J., Hernández-Hernández A., Llanillo M. (1998). Amphiphilic and hydrophilic forms of acetylcholinesterase from sheep platelets. *Biochimica et Biophysica Acta—Biomembranes*.

[30] Kaya H. B., Özcan B., Şişecioğllu M., Ozdemir H. (2013). Purification of acetylcholinesterase by 9-amino-1,2,3,4-tetrahydroacridine from human erythrocytes. *Applied Biochemistry and Biotechnology*.

[31] Sanchez-Yague J., Cabezas J. A., Llanillo M. (1990). Subcellular distribution and characterization of acetylcholinesterase activities from sheep platelets: relationships between temperature-dependence and environment. *Blood*.

[32] Vallee B. L., Ulmer D. D. (1972). Biochemical effects of mercury, cadmium and lead. *Annual Review of Biochemistry*.

[33] Cocco P. (1998). Occupational lead exposure and screening of glucose-6-phosphate dehydrogenase polymorphism: useful prevention or nonvoluntary discrimination?. *International Archives of Occupational and Environmental Health*.

